# Hyperpolarized Magnetic Resonance Imaging, Nuclear Magnetic Resonance Metabolomics, and Artificial Intelligence to Interrogate the Metabolic Evolution of Glioblastoma

**DOI:** 10.3390/metabo14080448

**Published:** 2024-08-14

**Authors:** Kang Lin Hsieh, Qing Chen, Travis C. Salzillo, Jian Zhang, Xiaoqian Jiang, Pratip K. Bhattacharya, Shyan Shams

**Affiliations:** 1 Department of Genitourinary Medical Oncology, The University of Texas MD Anderson Cancer Center, Houston, TX 77030, USA; khsieh@mdanderson.org; 2 Department of Computer Science, Louisiana State University, Baton Rouge, LA 70803, USAjz@lsu.edu (J.Z.); 3 Department of Radiation Oncology, The University of Texas MD Anderson Cancer Center, Houston, TX 77030, USA; 4 Department of Health Data Science and Artificial Intelligence, McWilliams School of Biomedical Informatics at UTHealth Houston, Houston, TX 77030, USA; 5 Department of Cancer Systems Imaging, The University of Texas MD Anderson Cancer Center, Houston, TX 77030, USA; pkbhattacharya@mdanderson.org

**Keywords:** artificial intelligence, HPMRI, PDX mice

## Abstract

Glioblastoma (GBM) is a malignant Grade VI cancer type with a median survival duration of only 8–16 months. Earlier detection of GBM could enable more effective treatment. Hyperpolarized magnetic resonance spectroscopy (HPMRS) could detect GBM earlier than conventional anatomical MRI in glioblastoma murine models. We further investigated whether artificial intelligence (A.I.) could detect GBM earlier than HPMRS. We developed a deep learning model that combines multiple modalities of cancer data to predict tumor progression, assess treatment effects, and to reconstruct in vivo metabolomic information from ex vivo data. Our model can detect GBM progression two weeks earlier than conventional MRIs and a week earlier than HPMRS alone. Our model accurately predicted in vivo biomarkers from HPMRS, and the results inferred biological relevance. Additionally, the model showed potential for examining treatment effects. Our model successfully detected tumor progression two weeks earlier than conventional MRIs and accurately predicted in vivo biomarkers using ex vivo information such as conventional MRIs, HPMRS, and tumor size data. The accuracy of these predictions is consistent with biological relevance.

## 1. Introduction

Glioblastoma multiforme (GBM), representing nearly half of all malignant brain and central nervous system tumor diagnoses, is typically first treated with surgical debulking. This is followed by six weeks of concurrent chemoradiotherapy with temozolomide, then six months of temozolomide chemotherapy. Unfortunately, despite this regimen, roughly 90% of GBM patients experience a recurrence, leading to a median survival duration of 8–16 months and a 5-year overall survival rate of 6.8% [1]. However, current GBM treatment cannot achieve satisfactory outcomes. Tan et al. [2] presented an overview of GBM treatments and suggested that precision biomarkers and an enhanced understanding of molecular biology may lead to more effective GBM therapies.

We propose that potential GBM progression biomarkers reside in the tumor’s cellular metabolism. A defining characteristic of cancer, the Warburg effect, involves cancer cells consuming and converting large amounts of glucose into lactate, even with sufficient oxygen for oxidative phosphorylation [2,3,4]. Positron emission tomography (PET) with 2-deoxy-2-[18F]fluoro- D-glucose (18F-FDG), which captures the first step of the Warburg effect, is a primary tool in cancer diagnosis and treatment follow-up. However, the utility of 18 F-FDG PET in brain tumor imaging is limited due to the high uptake of 18F-FDG by normal brain tissue. This limitation may be overcome with a new metabolic imaging modality known as hyperpolarized (HP) [1-13C]-pyruvate (13C-pyruvate) magnetic resonance (MR) imaging (MRI). Using dynamic nuclear polarization to amplify the MR signal of a 13C-pyruvate substrate [5], existing MR spectroscopy (MRS) and spectroscopic imaging methodologies can non-invasively image the uptake of an injected bolus of pyruvate and its subsequent conversion into lactate.

The positive outcomes of preclinical studies of HP 13C-pyruvate MRI in animal models led to various clinical trials of this technology for imaging GBM patients, particularly for monitoring treatment response. While these studies produced encouraging results, they only focused on one aspect of assessing GBM, such as detecting tumor growth or analyzing treatment response. In a recent study, we used longitudinal HP 13C-pyruvate MRI to measure metabolic changes in orthotopic murine models of GBM throughout tumor development, regression post-therapy, and recurrence. We also used longitudinal T2-weighted MRI to measure tumor volume, ex vivo nuclear MR (NMR) spectroscopy to measure steady-state metabolite pool size, and immunohistochemistry assays to measure protein expression. Our data analysis revealed that HP 13C-pyruvate MRS could consistently detect changes in tumor progression before significant changes in tumor anatomy occur [6]. This suggests that HPMRS could be utilized in clinical practice to predict tumor aggressiveness at diagnosis, differentiate pseudoprogression from real progression, predict patient survival after treatment, and/or identify an imminent relapse during follow-up. Our project’s comprehensive data indicated that using 13C-pyruvate MRS alone can identify tumor progression on Day 14 post-tumor cell implantation, seven days earlier than traditional MRI (21 Days). We then aimed to use radiomics and machine learning algorithms to enhance the detection of GBM progression.

HPMRS holds promise for early GBM diagnosis or predicting treatment effects, but it remains costly. Current clinical GBM diagnosis and treatment evaluation methods are based on histology and MRI imaging. By the time radiomic signals are strong enough to diagnose, patients are usually in the late stage of the disease. Artificial intelligence (A.I.) can provide an opportunity for early diagnosis. A.I. has shown utility in brain tumor segmentation, cancer prediction using blood metabolites, and other applications. It has also been used to integrate multiple types of information, such as clinical and genomic data, to predict long-term survival in cancer patients. Tumor progression data are distributed across various biomarkers and brain images. Learning the variation of these biomarkers and images can aid in predicting GBM progression, treatment effects, or related biomarkers.

We employed deep learning methods to address this complex situation, effectively integrating multiple signals across different modalities and time intervals. Our unique dataset for this project included metabolomic information and brain images across several time points, meeting our model needs. The limitation of the dataset is that only some mice have complete measurements at all time points. Given these circumstances, we applied deep learning technology to this unique dataset to answer three critical questions: (1) Can a multi-modality deep learning model predict tumor progression earlier than HPMRS? (2) Can the model predict or estimate the efficacy of a given therapy? (3) Can the model outline the key biochemical mechanisms leading to tumor progressions and therapeutic efficacies?

## 2. Material and Methods

### 2.1. Xenograft Mice

We used glioma sphere-forming cells (GSC) 8–11, obtained from a surgical sample provided by a female patient who gave written consent [7]. The University of Texas M. D. Anderson Cancer Center’s institutional review board approved the use of these cells, which are well-documented in existing literature. The cells were grown in Neurosphere Media, containing DMEM/F12 (Corning, Corning, NY, USA), B27 (×1, Thermo Fisher Scientific, Waltham, MA, USA), bFGF (20 ng/mL, Millipore Sigma, St. Louis, MO, USA), and EGF (20 ng/mL, Millipore Sigma, St. Louis, MO, USA). They were cultivated at 37 °C and authenticated by the MDACC Cell Authentication Core. Five-week-old athymic nude mice were used for in vivo studies. The mice were housed in a sterilized facility, with no more than five in a cage, and all were female to avoid fighting. They received standard feed and water, and their health was monitored daily. The intracranial xenografts followed the original literature’s procedures [8,9]. We suspended 5 × 10^5^ GSC 8–11 cells in 3 µL of phosphate-buffered saline (PBS) for the experiment group and injected them into the mice. The control mice received only PBS. All procedures complied with the University of Texas MD Anderson Cancer Center’s Institutional Animal Care and Use Committee (IACUC) regulations.

### 2.2. Mouse Cohorts

Following the intracranial implantation of patient-derived glioma sphere-forming cells (GSC), a variety of MRI sequences were employed to examine anatomic growth and shrinkage in vivo. These sequences included T1-weighted (T1-w), T2-weighted (T2-w), and fluid-attenuated inversion recovery (FLAIR). The real-time conversion of injected pyruvate to lactate within the tumor was measured in vivo using hyperpolarized 13C MRS. Both ex vivo metabolite pool sizes and protein expression were determined using NMR spectroscopy. Detailed methodologies for T1-w, T2-w, FLAIR, hyperpolarized 13C MRS, and NMR spectroscopy can be found in our previous paper [6]. The mice were divided into three cohorts [6]: untreated PDX-bearing control mice, treated PDX-bearing mice (undergoing 2 × 5 Gy radiotherapy), and untreated, non-PDX-bearing control mice. All procedures adhered to the regulations set by the Institutional Animal Care and Use Committee (00001263-RN02; 00001008-RN01) and the Institutional Review Board (LAB04-0001) of the University of Texas MD Anderson Cancer Center.

The study had three cohorts detailed below:

1. Untreated, non-PDX-bearing control mice (*n* = 27)
2. Untreated PDX-bearing mice (*n* = 67)
3. Treated PDX-bearing mice (*n* = 46) with 2 × 5 Gy of radiation daily on days 25 and 27.

### 2.3. Experimental Design

**Figure 1:**
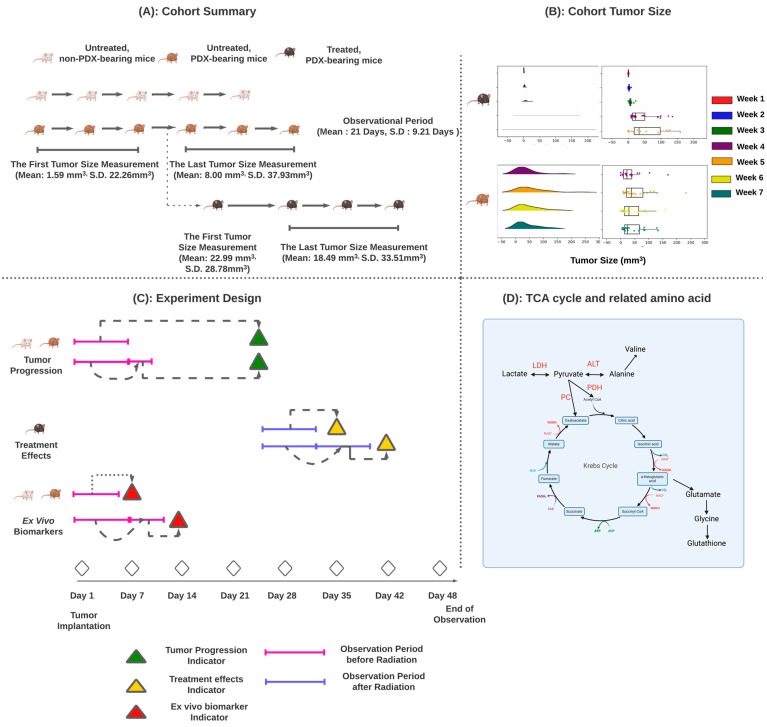
Overview of Cohorts and Tasks. (**A**) summarizes our cohort, tumor size, and tasks. The treated PDX-bearing mice are a subset of the untreated PDX-bearing mice. The first treated mouse was introduced on Day 21 after tumor implantation. (**B**) displays the tumor sizes for both untreated and treated PDX-bearing mice over several weeks as a rainfall plot. (**C**) lists the design of each experiment. We used tumor information from Day 1 to Day 14 to predict tumor progression after Day 21. This prediction involved identifying tumor progression using conventional MRI, HPMRS, and NMR biomarkers from untreated non-PDX-bearing mice and untreated PDX-bearing mice. The treatment effect prediction focused solely on treated PDX-bearing mice. We used conventional MRI, HPMRS, and NMR biomarkers after radiology to predict the outcome (tumor regression or progression) on the end date (Day 48). For predicting ex vivo biomarkers, we used conventional MRI and HPMRS to predict the status of ex vivo biomarkers (normal/abnormal). The tumor information was gathered before implementing the ex vivo biomarker measurement. (**D**) provides biological details of the TCA cycle and amino acid metabolism to understand the relationship between the in vivo biomarker (HPMRS) and ex vivo biomarker (NMR).

Figure 1 briefly summarizes the cohort and tasks. As mentioned earlier, the cohort consists of three different types of mice. However, not all mice underwent tumor measurement and MRI imaging at all time points due to the experimental design, resulting in some missing values. The first tumor size measurement for untreated, PDX-bearing mice was taken within two weeks of tumor implantation, with an average size of 1.59 mm^3^ and a standard deviation (S.D.) of 22.26 mm^3^. The final measurement was taken between Day 21 and Day 35 post-implantation, showing an average size of 8.00 mm^3^ and an S.D. of 37.93 mm^3^. A group of PDX-bearing mice received radiation treatment after Day 25. On Day 25, the average tumor size was 22.99 mm^3^ (S.D. 28.78 mm^3^). The final measurement for these mice was taken between Day 35 and Day 48 post-implantation, with an average size of 18.49 mm^3^ and an S.D. of 33.51 mm^3^. Additionally, Supplementary Figures S1–S3 provide more details. Supplementary Figure S1 displays raw MRI, segmentation, and tumor images, showing the overlap between the raw MRI and the segmentation across three plane views. Supplementary Figure S2 demonstrates the conversion pattern from C-13 pyruvate to lactate. Supplementary Figure S3 presents the tumor size after implantation. The blue color represents the untreated PDX-bearing mice, while the orange color signifies the treated PDX-bearing mice. Generally, the treated PDX-bearing mice exhibited a smaller tumor size compared to the untreated ones.

Our research aimed to determine three outcomes: (1) tumor progression, (2) treatment effects, and (3) ex vivo metabolomics. These tasks are detailed in the following section and summarized in Figure 1. Task one utilized tumor information, including anatomical MRI, HPMRS, tumor size measurements, and ex vivo biomarkers up to the point of tumor progression for each mouse. This task involved two cohorts: (1) untreated, non-PDX-bearing mice and (2) untreated, PDX-bearing mice. The treatment effects task used the same data input as the tumor progression task but focused only on treated PDX-bearing mice. For task three, we excluded ex vivo biomarkers from the data input, focusing solely on untreated, non-PDX-bearing mice and untreated, PDX-bearing mice. This task aimed to establish a correlation between the in vivo and ex vivo biomarkers for tumor progression. The correlation between ex vivo and in vivo biomarkers is strongly related to the TCA cycle. We further listed the amino acid metabolism associated with the TCA cycle as in Figure 1. As shown, pyruvate can be converted into alanine and subsequently into valine. Other amino acids, such as glutamate, glycine, and glutathione, can derive from alpha-ketoglutaric acid. Lastly, the details of the number of mice used in each experiment are listed in Table 1.

### 2.4. Tumor Progression

Following tumor implantation, GBM tumor cells infiltrate surrounding brain regions, which gradually increases the tumor size. Once the tumor is sufficiently large, it can be detected by MRI for standard treatments such as surgery. In previous studies, untreated tumors developed from the time of implantation. Treatment was administered on Days 25 and 27 post-implantation, with ongoing monitoring of tumor size. The average initial tumor volume at this point was 26 mm^3^. We considered a tumor volume greater than 25 mm^3^ as an indicator of tumor progression. Therefore, our model should identify tumor progression before the first record of such progression in the cohort. The first mice presenting a tumor volume above 25 mm^3^ were identified on Day 21. Consequently, we discarded data measured between Day 21 and Day 28. The task is to predict tumor progression between Day 21 and Day 28, using only the cancer information from Day 1 to Day 24. We established three-time points before Day 21 to assess whether temporal information (multiple measurements of a mouse) can enhance model performance. The tumor progression dataset included untreated, non-PDX-bearing control mice and untreated PDX-bearing mice.

### 2.5. Treatment Effects

After radiation therapy, the tumor volumes began to regress. In previous research, we identified the tumor regression period as Day 25–48, which represents the time between treatment and the point when the average tumor size started to increase due to relapse. We created a model that uses this period’s tumor information to predict the efficacy of radiation therapy. We defined effective radiation therapy as follows:

1. Effective radiation therapy should result in a reduction in tumor size over the tumor regression period. This reduction is indicated by a negative correlation between day and tumor volume. We classified mice with a negative correlation as having a successful treatment response and vice versa.
2. If a mouse only had one tumor measurement within the tumor regression period, we established a reference line from untreated PDX-bearing mice. This line provides an estimate of tumor size in the event of treatment failure. We believe that effective treatment should result in a tumor size smaller than the lower-bound reference line. We used a generalized linear model to construct this line, which represents the estimated average tumor volume minus one standard deviation of the estimated tumor volume. If a mouse’s tumor volume falls below this line, radiation therapy is considered effective.

For this task, the dataset only includes treated PDX-bearing mice. We designated Day 28 as Day 1 post-radiation therapy.

### 2.6. Ex Vivo Metabolomic Prediction

Our goal was to evaluate the model’s ability to use in vivo data to predict ex vivo results. We used anatomical MR images, tumor volume measurements, and HPMRS data to predict NMR spectroscopy-measured biomarker levels. These biomarkers include those related to (1) amino acid metabolism (valine, alanine, and glycine), (2) the cell membrane (glycerophosphocholine, phosphocholine, and phosphoethanolamine), and (3) reactive oxygen species (glutathione and nicotinamide adenine dinucleotide). We established the normal range for each biomarker by calculating the average and standard deviation from untreated, non-PDX-bearing mice. We then set a threshold using Mean±S.D. to identify abnormal levels for each NMR biomarker. If an NMR biomarker’s value exceeded this threshold, it was deemed abnormal due to an exceed-normal range from untreated, non-PDX-bearing mice.

### 2.7. Model Design

This model is the first, according to available data, to utilize anatomical MRI, HPMRS, and NMR for task execution. Given the complexity of the data, the model employs two separate encoders to derive features from high-dimensional tumor information like anatomical MRI and HPMRS, subsequently creating low-dimensional embeddings. For instance, the MRI processing unit incorporates data from various planes and time points from T2-weighted MRI. Likewise, the HPMRS processing unit generates a comprehensive tumor representation using data from multiple time points. When combined with additional tabular data, such as ex vivo NMR metabolomics data, these representations enable the model to make its final prediction. We will illustrate the function of each component within the model.

### 2.8. Model Components

**Figure 2:**
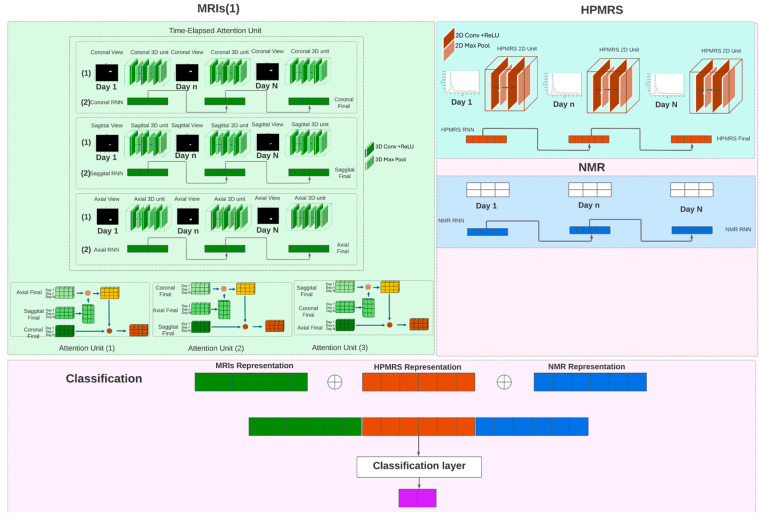
Overview of the model. Given the complexity of the dataset, we created a model with two encoders to extract the feature map from MRI images and HPMRS. The input data for MRI images are the segmented tumor regions, while real-time pyruvate to lactate transformations serve as input for HPMRS. The model generates three types of temporal representation from MRI images (1), HPMRS (2), and NMR information (3). Then, these representations are combined for the final prediction. The MRI processing unit includes an MRI encoder and a time-elapsed attention module. The MRI encoder contains three 3D CNNs, each followed by a 3D max-pooling layer. The kernel sizes for the first, second, and third CNN/max-pooling layer pairs are (2, 3, 3), (1, 3, 3), and (1, 3, 3), respectively. The output channel sizes for the three CNNs are 64, 128, and 256, in that order. Each 3D image encoder generates a representation of a specific day from a given plane. Each mouse has three matrices to represent the axial, sagittal, and coronal planes. The matrix for each plane type is M∈ of RD × l. ‘D’ ranges from Day 1 to a certain day (e.g., Day 3–Day 14), and ‘l’ is the representation length (size 128). Each plane matrix first undergoes a self-attention module, then is processed by multi-head attention (with a head number of 2) to obtain the final image representation. The HPMRS processing unit includes an HPMRS encoder and a recurrent neural network (RNN). Each HPMRS encoder processes HPMRS of a given time point (e.g., Day 8, Day 14). The RNN sequentially learns the representation from each HPMRS encoder and generates HPMRS representation. The HPMRS encoder contains two 2D CNNs, each followed by a 2D max-pooling layer. Both the first and last CNN/max-pooling layer pairs have a kernel size of (3, 3). The output channel sizes for the first and last CNNs are 8 and 16, respectively. The RNN in the HPMRS encoder only has one layer.

As shown in Figure 2, our model is composed of four parts to handle different modalities. The first part, the MRIs processing unit, includes the MRIs encoder and the time-elapsed attention unit. The HPMRS images are processed by a unique module called the HPMRS processing unit, which consists of the HPMRS encoder and a recurrent neuron network (RNN). The remaining NMR information is processed using another RNN. The final part of the model, the classifier, combines all the representations from each unit to make its prediction. We provide more details about the model in Table 2.

### 2.9. MRIs Processing Unit

The processing unit is composed of 2 major modules: (1) a 3-dimensional (3D) image encoder, which generates feature maps from 3D MRIs, and (2) a time-elapsed attention module to select the best feature maps for classification purposes.

#### 2.9.1. MRIs Encoder

Figure 2 shows the design of the 3D image encoder. T2-weighted MRI images containing spatial and textural information can be used to detect GBM invasion [10]. To capture spatial relationships among different MRI slices, we use a 3D convolutional neural network [11] (CNN). Equation (1) represents the 3D convolution layer that extracts the feature map (*Z_l_*) from T2-weighted MRI images. These feature maps (*Z_l_*) are further processed by the ReLU function, as described in Equation (2). The activated feature maps (*Z_l_*) are then processed by the maxpooling layer, as described in Equation (3).

(1) $${\left(Z_{l}\right)}_{ijkm}=\sum_{c=1}^{C}\sum_{u=0}^{U-1}\sum_{v=0}^{V-1}\sum_{w=0}^{W-1}{\left(W_{l}\right)}_{uvwcm}{\left(X_{l-1}\right)}_{\left(i+u\right)\left(j+v\right)\left(k+w\right)c}+{\left(B_{l}\right)}_{m}$$

(2) $${\left(Z_{l}\right)}_{ijkm}=\mathrm{ReLU}\left(Z_{ijkm}\right)=\max\left(0,{\left(Z_{l}\right)}_{ijkm}\right)$$

(3) $${\left(Z_{l}\right)}_{ijkm}={\underset{u,v,w}{\max}\left(Z_{l}\right)}_{\left(i+u\right)\left(j+v\right)\left(k+w\right)m}$$

Our model processes data at multiple time points, from Day 1 to Day N, which results in a set of feature maps for the same period. We denote this set as $I=\left\{Z_{l1^{,}}Z_{l2^{\ldots \ldots .}}Z_{lN}\right\}$, representing the tumor progression feature map over time. To handle the tumor progression at each time point, we use a recurrent neural network (RNN), the equations of which are detailed in Equations (4) and (5). Finally, we derive three other sets $La=\left\{l_{a1^{,}}l_{a2^{\ldots \ldots }}l_{aN}\right\}$; $Lc=\left\{l_{c1^{,}}l_{c2^{\ldots \ldots }}l_{cN}\right\}$; $Ls=\left\{l_{s1}l_{s2^{\ldots \ldots }}l_{sN}\right\}$ to represent individually the axial view, coronal view, and sagittal view across different time points.

(4) $$h_{t}=\sigma_{h}\left(W_{h}Z_{lt}+U_{h}Z_{lt-1}+b_{h}\right)$$

(5) $$y_{t}=\sigma_{y}\left(W_{y}h_{t}+b_{y}\right)$$

#### 2.9.2. Time-Elapsed Attention Module

Tumor progression is reflected in anatomic MRI across both spatial (axial, coronal, and sagittal image planes) and temporal (days after tumor implantation) dimensions. As discussed earlier, we use $La=\left\{l_{a1^{,}}l_{a2^{\ldots \ldots }}l_{aN}\right\}$; $Lc=\left\{l_{c1^{,}}l_{c2^{\ldots \ldots }}l_{cN}\right\}$; $Ls=\left\{l_{s1}l_{s2^{\ldots \ldots }}l_{sN}\right\}$ to represent these dimensions separately. Time is significant due to the elapsed days after tumor implantation. This led us to develop a time-elapsed attention model to understand the temporal interaction among different time points, represented by Equation (6). The time-elapsed attention module consists of two parts: (1) a self-attention module [12], identifying contextual information within, such as the relationship between the axial representation on Day 1 and Day 2, and (2) a multi-head attention module [13], capturing the contextual information between different views (e.g., axial vs. sagittal, sagittal vs. coronal). We swapped the query, key, and value for the second module to derive a more comprehensive representation from MRIs. The query, key, and value are represented by different plane views. We finally concatenated three representations (axial, sagittal, coronal) into a single vector to get the final representation. The details of the process are presented in Figure 2.

(6) $$\mathrm{Attention}\left(Q,K,V\right)=\mathrm{softmax}\left(\frac{QK^{T}}{\sqrt{d_{k}}}\right)V$$

### 2.10. HPMRS Processing Unit

Hyperpolarized magnetic resonance spectroscopy (HPMRS) is a 2-D data recording system that collects intensity and time points. The intensity represents the real-time transformation from pyruvate to lactate, recorded over a period. To manage this unique data type, we developed an HPMRS processing unit. Each HPMRS at a specific time was initially processed using a sequence. This sequence included a 2D convolution layer, a rectified linear unit (ReLU), and a 2D maxpooling layer. We used this sequence to derive a representation for a given time point (e.g., Day 1). Afterwards, we processed the cross-time point HPMRS representation with an RNN to further derive the temporal representation. We used this representation to combine other MRI and NMR representations for the final prediction. The details of the process are presented in Figure 2.

### 2.11. Classifier

All data obtained from MRI, HPMRS, and NMR are combined and processed through a binary classifier. This classifier calculates the likelihood of the input data belonging to a specific class for each task. The definitions of “positive class” and “negative class” are dependent on the experiment. The loss function is outlined in Equation (7), where *x* represents the predicted probability and *y* denotes the true label.

(7) $$l=-w\left[y\times \log\left(x\right)+\left(1-y\right)\times \log\left(1-x\right)\right]$$

### 2.12. Training and Evaluation

We performed repeated k-fold cross-validation on each task. For each task, we used 5 epochs, with each epoch containing 3 folds. In each fold, the ground truth labels were withheld in the test data, and the classification accuracy was measured using the area under the receiver operating characteristic curve (AUC), true positive rate vs. false positive rate (AUPRC), true-positive rate (TPR), false-negative rate (FNR), false-positive rate (FPR), and true-negative rate (TNR).

### 2.13. Missing Value Handling

To minimize the impact of missing values, we ensured that mice had at least one anatomical MRI and/or one HPMRS measurement. We also assumed that mice within the same cohort had similar conditions. Thus, we substituted missing values with the average values from the same cohort on the same day. However, these imputed data were masked during computation, indicating they did not affect the final results.

## 3. Results

### 3.1. Prediction of Tumor Progression

**Figure 3:**
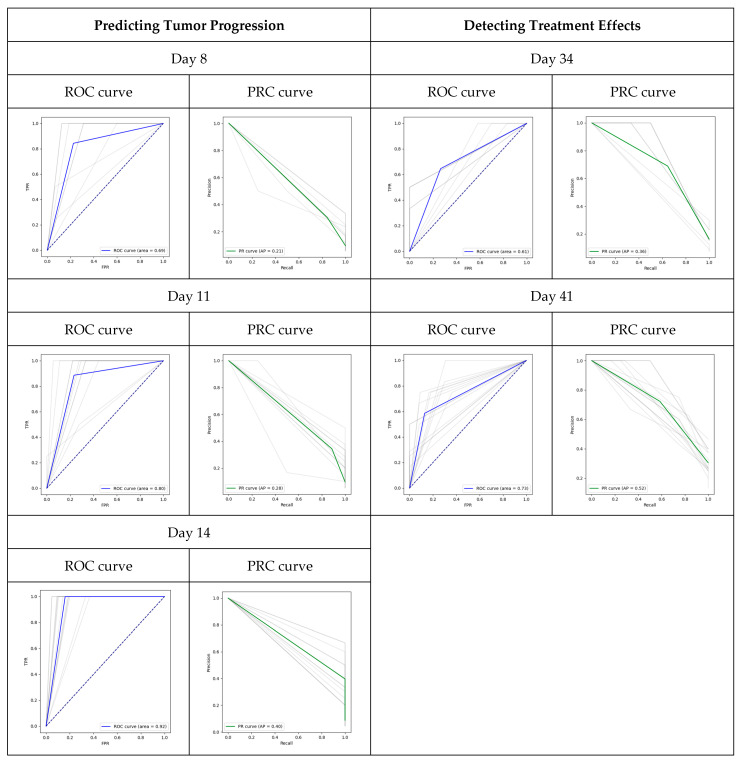
ROC curve and PRC curve for both predicting tumor progression and detecting treat-ment effects. We presented both the AUROC and AUPRC for two tasks: (1) predicting tumor progression and (2) detecting treatment effects. Adding temporal information was observed to enhance the model’s performance, particularly in predicting tumor progression. In Figure 3, each experiment’s ROC curve and PRC curve are plotted as grey lines, with the blue line indicating the average ROC curve and the green line indicating the average PRC curve.

The model’s performance in predicting tumor progression before Day 28 is detailed in Table 3. As early as Day 7, the model predicted tumor progression with an average AUC of 0.69. By Day 14, the average AUC value improved to 0.919, indicating that the incorporation of temporal information can enhance the model’s performance. Furthermore, the reduction of the standard deviation (S.D.) of AUC from Day 7 to Day 14 suggests that adding temporal information can also improve the model’s generalizability. We calculated the true positive rate (TPR), false negative rate (FNR), false positive rate (FPR), and true negative rate (TNR) for both the non-PDX-bearing mice and untreated PDX-bearing mice. Since the non-PDX-bearing mice did not possess PDX tumors, they should not have shown any signs of tumor progression. The mean tumor-to-normal ratio (TNR) was 0.95, and the false positive rate (FPR) was 0.05 across different time points. The true positive rate (TPR) and false negative rate (FNR) were both 0.0 at different times, indicating the model’s high accuracy. For untreated PDX-bearing mice, the TPR increased from 0.52 on Day 7 to 1.0 on Day 14, reinforcing the belief that temporal information enhances the model’s performance. In general, the model demonstrated high specificity but relatively low sensitivity. Both the ROC curve and PRC cure are also presented in Figure 3. As shown in Figure 3, adding temporal information reduces the variation of AUROC, making the AUROC of each experiment closer to the average AUROC. Similar patterns are observed in AUPRC.

### 3.2. Detection of Treatment Efficacy

The model’s performance in predicting treatment effects is outlined in Table 4. Seven days post-treatment, the area under the curve (AUC) registered at 0.608, increasing to 0.728 by day fourteen, as indicated by cross-validation. However, there was a decrease in AUC variation over time. The true positive rate (TPR) increased from 0.369 to 0.585 between the seventh and fourteenth day, while the false positive rate (FPR) decreased from 0.151 to 0.129 over the same period. All benchmark variations also showed a decrease over time. These trends indicate that adding temporal information could slightly improve the model’s performance. The ROC and PRC curves are displayed in Figure 3. We also observed that adding temporal information can reduce the variation in detecting treatment effects. However, the variation remains larger than predicting tumor progression.

### 3.3. Prediction of Biomarkers Ex Vivo

**Figure 4:**
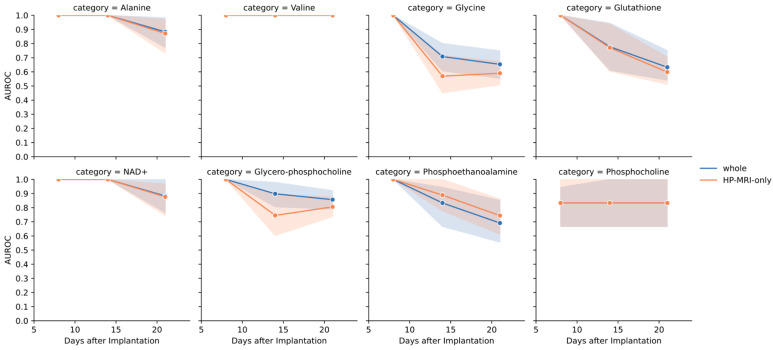
Prediction of ex vivo biomarkers. In Figure 4, our results showed that the AUROC of the amino acid metabolism group, which includes alanine, valine, and glycine, outperformed other biomarkers. For most biomarkers, using only HPMRS data yielded similar performance as using the full information. However, the values of glycine and glycero-phosphocholine decreased by more than 10% when only HPMRS data was used.

The model predicts whether a given biomarker is normal or abnormal on Days 8, 14, and 21. We compared the model’s predictions with the actual results using AUC. The model was generally more accurate in predicting the status of amino acid metabolism biomarkers compared to reactive oxygen metabolism biomarkers or cell membrane metabolism biomarkers (Figure 4). To determine the contribution of HPMRS to the prediction of ex vivo biomarkers, we excluded anatomical MRI data and used only HPMRS information. The accuracy of most biomarkers remained similar when using this complete information. However, two ex vivo biomarkers, glycine and glycero-phosphocholine, dropped by more than 10%. This suggests that the model’s prediction of amino acid metabolism is more reliable than its prediction of the other two categories. We also presented the AUROC and AUPRC of each biomarker across different time points in Supplementary Table S1.

## 4. Discussion

Our model demonstrated promising performance in predicting tumor progression and identifying ex vivo biomarkers. However, it had limited performance in predicting treatment effects. The model could detect tumor progression as early as seven days after implanting GSC 8–11, a full week earlier than using HPMRS [6]. Both the AUC and sensitivity increased over time until Day 14. Similar patterns were observed to evaluate treatment effects, with AUC rising over time in predicting tumor progression. These findings imply that adding temporal information improves the model’s performance. In addition, adding temporal information can reduce the variation of both AUROC and AUPRC.

### 4.1. Temporal Patterns Improve Model Performance

In an experimental setting, obtaining different types of measurements across multiple time points for all mice is impossible. For instance, once a mouse is humanely euthanized for ex vivo NMR analysis, it cannot be used for subsequent HPMRS experiments. Thus, a high proportion of missing values for each mouse presented the biggest challenge in this study. We assumed that analyses of MR images, NMR measurements, and HPMRS results would reveal similar tumor progression patterns for mice in the same cohort. Temporal cycle-consistency learning [14] uses different videos with the same sequential action to learn each video frame over time. The results showed that A.I. can align various sources of video frames with the same sequence action. This implies that the A.I. model can learn from different mice within the same cohort and still incorporate temporal tumor information into the model. Despite the dataset having a high proportion of missing values, this assumption was validated as the model’s performance in tumor progression and treatment effects improved over time.

### 4.2. Deep Learning Can Be Used to Predict Treatment Effects in Preclinical Models of GBM

Our model demonstrates the potential of A.I. to expedite the assessment of treatment effects in GBM patients. The model produced a satisfactory AUC even with a limited number of mice. The AUC improved over time, suggesting that temporal information could enhance the model’s performance. Furthermore, the use of temporal patterns boosted the TPR from 0.369 to 0.585 and reduced the FPR from 0.151 to 0.129.

### 4.3. Deep Learning Can Be Combined with HPMRS to Predict Metabolomic Patterns

Our model discovered associations between in vivo MRI and ex vivo HPMRS biomarkers, as measured by NMR. The model can predict biomarkers of amino acid metabolism more accurately than it can predict two other types of biomarkers: cell membrane and reactive oxygen species. The results inferred some biological mechanisms. For instance, the HPMRS probe 13C-pyruvate is employed to detect real-time glycolysis in vivo [15]. However, glycolysis can only generate two ATP molecules per pyruvate. Alternative energy sources likely need to be used for ATP production and cell building blocks [4]. Potential sources could include fatty acid oxidation and the Cahill cycle, which convert various metabolites into tricarboxylic acid cycle intermediates, as well as one-carbon metabolism, which contributes to cellular biomass production [16]. The conversion rate of HP 1-13C pyruvate to HP 1-13C lactate depends on the relative concentration of endogenous pyruvate and lactate pool sizes, as well as the lactate dehydrogenase enzyme. These factors can vary with the rate of nearby metabolic processes and the cell’s redox state [17]. Therefore, we reasonably infer that the kinetics of HP 1-13C pyruvate to HP 1-13C lactate conversion measured in HPMRS experiments could predict other metabolic processes in the cell. We observed this phenomenon in our study with respect to amino acid metabolism. While these findings are still preliminary, we believe that continual exploration of these relationships could potentially lead to biopsy-free metabolomics. This would be especially useful for longitudinal metabolic examination in clinical scenarios, such as monitoring treatment responses, and for solid tumors in hard-to-reach areas like the brain.

## 5. Limitations

The study’s main limitations were the small sample size and the high proportion of missing values. While this can affect the generalizability of the model, the initial result in this pilot study is promising and warrants further research and study in applying AI to multi-modal data. In addition, the current model was only examined at individual time points and lacked evaluation at multiple time points due to the sample size. Once we accumulate enough examples, we will try to develop a temporal projection model.

## 6. Conclusions

In this study, we developed a multi-modality deep learning model that integrated multiple types of tumor information to predict tumor progression, evaluate treatment effects, and transform in vivo information into ex vivo information. The findings suggest that the model can identify tumor progression as early as 7 days after tumor implantation, a duration that is considerably shorter than that achievable with HPMRS only (14 days after tumor implantation) based on our previous research [6]. The model also could predict treatment effects and identify biological correlations between the HPMRS probe ^13^C-pyruvate and its related metabolites. HP ^13^C-pyruvate MRI, despite its strengths in real-time metabolic imaging, can detect only one or two metabolites in vivo. Our predictive model has the potential to expand the number of metabolites that can be interrogated in an in vivo study.

## Figures and Tables

**Table 1:** Number of mice used to train and test the model for each experiment.

No. of Days after Index Date or Treatment	Cohort	No. of Mice
Predicting Tumor Progression		
8	Control	10
	Untreated	42
11	Control	13
	Untreated	47
14	Control	17
	Untreated	51
Predicting Treatment Effects		
7	Treated	39
14	Treated	46
Predicting in vivo biomarkers		
8	Control	3
	Untreated	7
14	Control	9
	Untreated	17
21	Control	12
	Untreated	22

**Table 2:** Parameters of the model.

Modula_Name	Kernel Size	Output Size	Number of Parameters
Fianl Model		[4, 2]	42,304,277
Sagittal_3D_Encoder-1		[4, 64, 3, 35, 48]	1216
Conv3d: 2	[2, 3, 3]	[4, 64, 6, 105, 145]	1216
ReLU: 2		[4, 64, 6, 105, 145]	0
MaxPool3d: 2	[2, 3, 3]	[4, 64, 3, 35, 48]	0
Sagittal_3D_Encoder-2		[4, 128, 3, 11, 15]	73,856
Conv3d: 2	[1, 3, 3]	[4, 128, 3, 33, 46]	73,856
ReLU: 2		[4, 128, 3, 33, 46]	0
MaxPool3d: 2	[1, 3, 3]	[4, 128, 3, 11, 15]	0
Sagittal_3D_Encoder-3		[4, 256, 3, 3, 4]	295,168
Conv3d: 2	[1, 3, 3]	[4, 256, 3, 9, 13]	295,168
ReLU: 2		[4, 256, 3, 9, 13]	0
MaxPool3d: 2	[1, 3, 3]	[4, 256, 3, 3, 4]	0
Axial_3D_Encoder-1		[4, 64, 2, 35, 48]	1216
Conv3d: 2	[2, 3, 3]	[4, 64, 5, 105, 145]	1216
ReLU: 2		[4, 64, 5, 105, 145]	0
MaxPool3d: 2	[2, 3, 3]	[4, 64, 2, 35, 48]	0
Axial_3D_Encoder-2		[4, 128, 2, 11, 15]	73,856
Conv3d: 2	[1, 3, 3]	[4, 128, 2, 33, 46]	73,856
ReLU: 2		[4, 128, 2, 33, 46]	0
MaxPool3d: 2	[1, 3, 3]	[4, 128, 2, 11, 15]	0
Axial_3D_Encoder-3		[4, 256, 2, 3, 4]	295,168
Conv3d: 2	[1, 3, 3]	[4, 256, 2, 9, 13]	295,168
ReLU: 2		[4, 256, 2, 9, 13]	0
MaxPool3d: 2	[1, 3, 3]	[4, 256, 2, 3, 4]	0
Coronal_3D_Encoder-1		[4, 64, 4, 35, 48]	1216
Conv3d: 2	[2, 3, 3]	[4, 64, 8, 105, 145]	1216
ReLU: 2		[4, 64, 8, 105, 145]	0
MaxPool3d: 2	[2, 3, 3]	[4, 64, 4, 35, 48]	0
Coronal_3D_Encoder-2		[4, 128, 4, 11, 15]	73,856
Conv3d: 2	[1, 3, 3]	[4, 128, 4, 33, 46]	73,856
ReLU: 2		[4, 128, 4, 33, 46]	0
MaxPool3d: 2	[1, 3, 3]	[4, 128, 4, 11, 15]	0
Coronal_3D_Encoder-3		[4, 256, 4, 3, 4]	295,168
Conv3d: 2	[1, 3, 3]	[4, 256, 4, 9, 13]	295,168
ReLU: 2		[4, 256, 4, 9, 13]	0
MaxPool3d: 2	[1, 3, 3]	[4, 256, 4, 3, 4]	0
HPMRS_2D_Encoder-1		[4, 8, 5, 843]	80
Conv2d: 2	[3, 3]	[4, 8, 16, 2530]	80
ReLU: 2		[4, 8, 16, 2530]	0
MaxPool2d: 2	[3, 3]	[4, 8, 5, 843]	0
HPMRS_2D_Encoder-2		[4, 16, 1, 280]	1168
Conv2d: 2	[3, 3]	[4, 16, 3, 841]	1168
ReLU: 2		[4, 16, 3, 841]	0
MaxPool2d: 2	[3, 3]	[4, 16, 1, 280]	0
Time-Elapsed Attention:1		[4, 768]	786,432
Axial Attention: 1		[1, 1, 256]	262,144
ReLU: 2		[1, 4, 256]	0
Coronal Attention: 1		[1, 1, 256]	262,144
Sagittal Attention: 1		[1, 1, 256]	262,144
ReLU: 2		[4, 768]	0
Time-Elapsed Attention:2		[4, 768]	786,432
Coronal Attention: 2		[1, 1, 256]	262,144
ReLU: 2		[1, 4, 256]	0
Axial Attention: 2		[1, 1, 256]	262,144
Sagittal Attention: 2		[1, 1, 256]	262,144
ReLU: 2		[4, 768]	0
Time-Elapsed Attention:3		[4, 768]	786,432
Sagittal Attention: 3		[1, 1, 256]	262,144
ReLU: 2		[1, 4, 256]	0
Coronal Attention: 3		[1, 1, 256]	262,144
Axial Attention: 3		[1, 1, 256]	262,144
ReLU: 2		[4, 768]	0
Classification: 1		[4, 768]	1,770,240
ReLU: 1		[4, 768]	0
NMR_RNN: 1		[4, 1, 9]	180
HP_RNN: 1		[4, 1, 128]	52,480
Classification: 2		[4, 2]	1818

**Table 3:** Performance of identifying tumor progression.

**Overall** **(Mean** **/S** **.D** **.)**					
**Day**		**AUC**		**AUPRC**	
7		0.690/0.184		0.211/0.086	
11		0.799/0.145		0.279/0.094	
14		0.919/0.049		0.396/0.174	
**By Cohort (Mean** **/S.D.)**					
**Day**	**Cohort**	**TPR**	**FNR**	**FPR**	**TNR**
7	non-PDX-bearing mice	0/0	0/0	0.052/0.184	0.948/0.184
7	untreated PDX-bearing mice	0.519/0.483	0.480/0.483	0.159/0.201	0.840/0.201
11	non-PDX-bearing mice	0/0	0/0	0.059 ± 0.150	0.941/0.150
11	untreated PDX-bearing mice	0.812/0.355	0.187/0.355	0.261/0.175	0.738/0.175
14	non-PDX-bearing mice	0/0	0/0	0.059/0.069	0.941/0.150
14	untreated PDX-bearing mice	1/0	0/0	0.206/0.105	0.793/0.105

**Table 4:** Performance of detecting treatment effects.

**Overall** **(Mean** **/S** **.D** **.)**					
**Day**		**AUC**		**AUPRC**	
7		0.608/0.607		0.356/0.166	
14		0.728/0.081		0.520/0.107	
**By Cohort (Mean** **/S.D.)**					
**Day**	**Cohort**	**TPR**	**FNR**	**FPR**	**TNR**
7	Treated	0.369/0.398	0.630/0.398	0.151/0.304	0.848/0.304
14	Treated	0.585/0.244	0.414/0.244	0.129/0.126	0.870/0.126

## Data Availability

The Data and Code of this study can be downloaded from https://github.com/freshnemo/HPMRI (accessed on 1 July 2024).

## Supplementary Materials

The following supporting information can be downloaded at: https://www.mdpi.com/article/10.3390/metabo14080448/s1, Figure S1: One Example of Mice MRI Images on Different Planes; Figure S2: One Example of HPMRS shows Pyruvate (colored in blue) converting to Lactate (colored in orange); Figure S3: Tumor size measurement overtime after tumor implantation; Table S1: Metabolite Prediction Summary Table.
