# Supplementary material for: Hyperpolarized Magnetic Resonance Imaging, Nuclear Magnetic Resonance Metabolomics, and Artificial Intelligence to Interrogate the Metabolic Evolution of Glioblastoma
Source: Metabolites. 2024 Aug 14;14(8):448. doi: 10.3390/metabo14080448 (PMC11356718; doi:10.3390/metabo14080448)

Supplementary Table
Metabolite Prediction Summary Table

Biomarkers Category	Biomarkers	Days after tumor Implantation	AUROC (Mean±SD)	AUPRC (Mean±SD)
Amino acid metabolism	Alanine	8	1.0	1.0
Amino acid metabolism	Alanine	14	1.0	1.0
Amino acid metabolism	Alanine	21	1.0	1.0
Amino acid metabolism	Valine	8	1.0	1.0
Amino acid metabolism	Valine	14	1.0	1.0
Amino acid metabolism	Valine	21	1.0	1.0
Amino acid metabolism	Glycine	8	1.0	1.0
Amino acid metabolism	Glycine	14	0.63±0.20	0.32±0.27
Amino acid metabolism	Glycine	21	0.62±0.15	0.24±0.11
Reactive oxygen metabolism	NAD+	8	1.0	1.0
Reactive oxygen metabolism	NAD+	14	1.0	1.0
Reactive oxygen metabolism	NAD+	21	1.0	1.0
Reactive oxygen metabolism	Glutathione	8	1.0	1.0
Reactive oxygen metabolism	Glutathione	14	0.77±0.26	0.62±0.42
Reactive oxygen metabolism	Glutathione	21	0.61±0.16	0.24±0.28

cell membrane metabolism	Glycerol-Phosphocholine	8	1.0	1.0
cell membrane metabolism	Glycerol-Phosphocholine	14	0.82±0.20	0.66±0.33
cell membrane metabolism	Glycerol-Phosphocholine	21	0.83±0.11	0.62±0.23
cell membrane metabolism	Phosphocholine	8	0.83±0.24	0.77±0.32
cell membrane metabolism	Phosphocholine	14	0.83±0.24	0.70±0.42
cell membrane metabolism	Phosphocholine	21	0.83±0.24	0.69±0.44
cell membrane metabolism	Phospho-ethanoalamine	8	1.0	1.0
cell membrane metabolism	Phospho-ethanoalamine	14	0.86±0.23	0.75±0.40
cell membrane metabolism	Phospho-ethanoalamine	21	0.71±0.22	0.48±0.40

Supplementary Figures

Figure 1: One Example of Mice MRI Images on Different Planes

**Saggital View**		
**Raw MRI**	**Segmentation**	**Tumor**
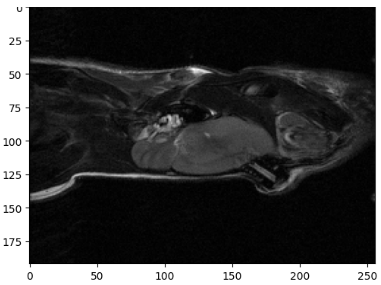	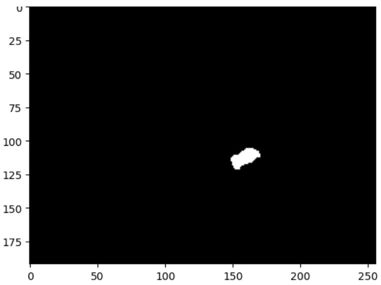	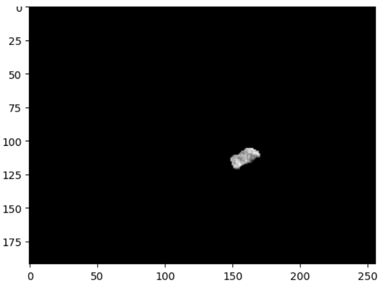

Axial View

Raw MRI	Segmentation	Tumor

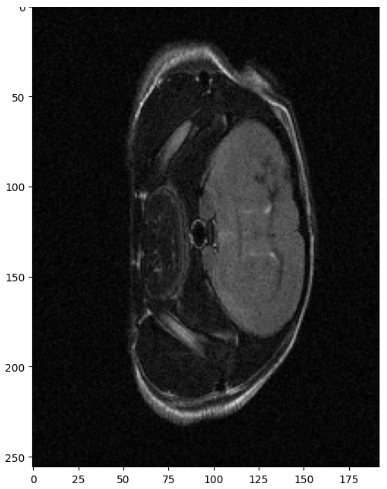

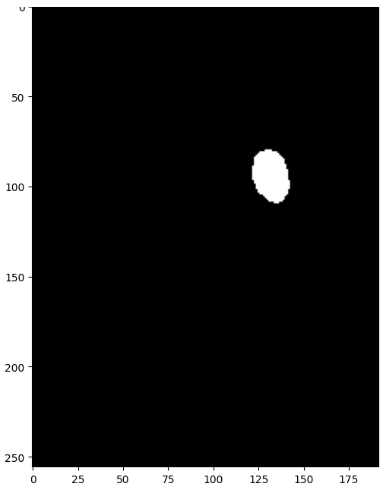

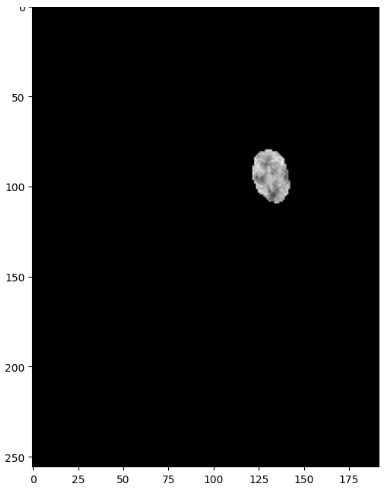

Coronal View

Raw MRI	Segmentation	Tumor

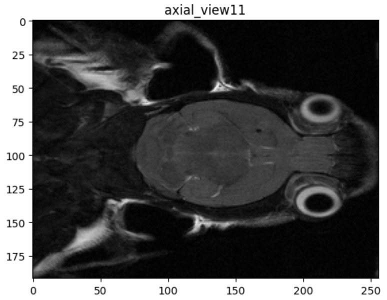

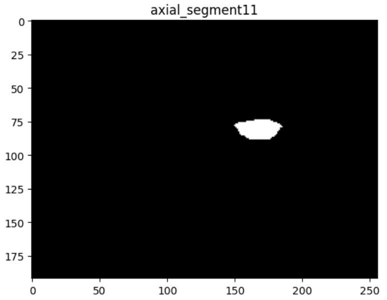

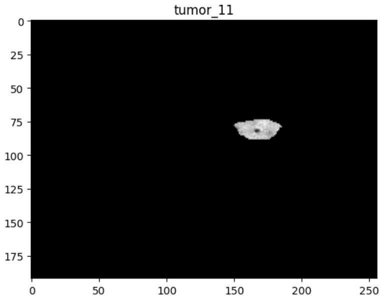

Figure 2: One Example of HPMRS shows Pyruvate (colored in blue) converting to Lactate (colored in orange)

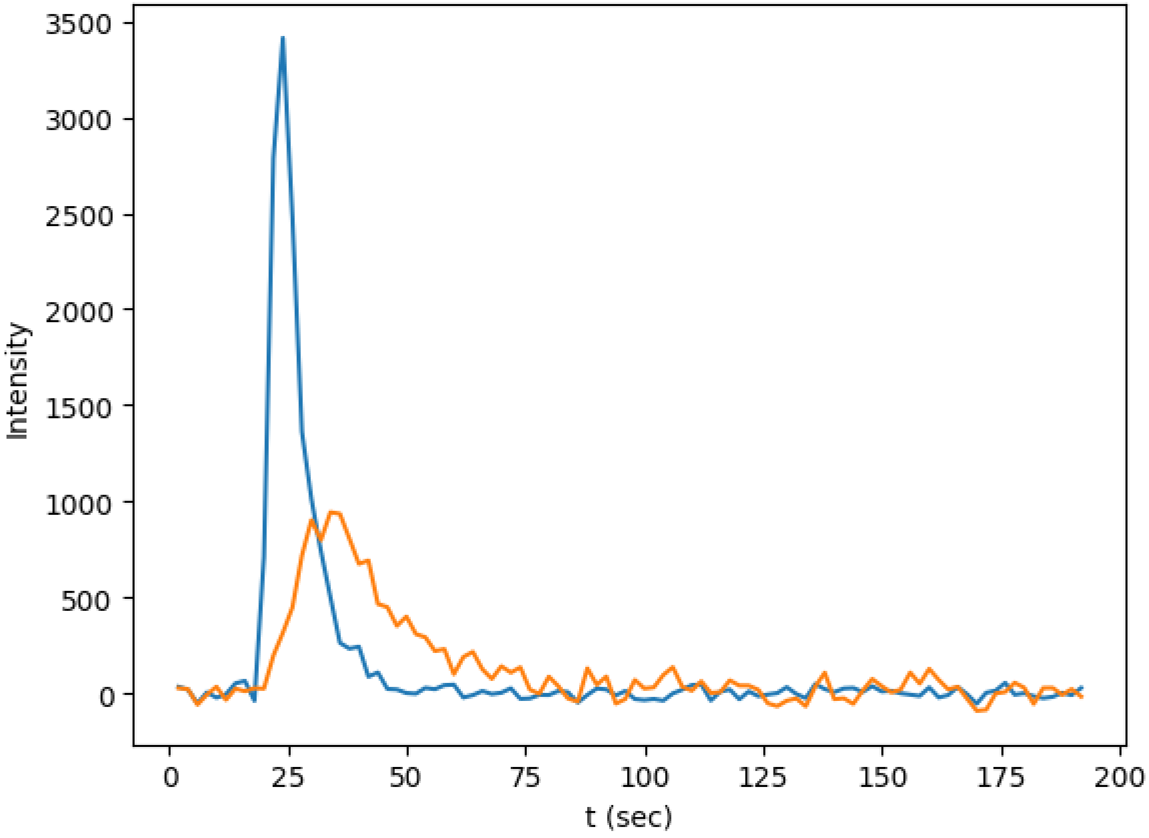

Figure 3: Tumor size measurement overtime after tumor implantation

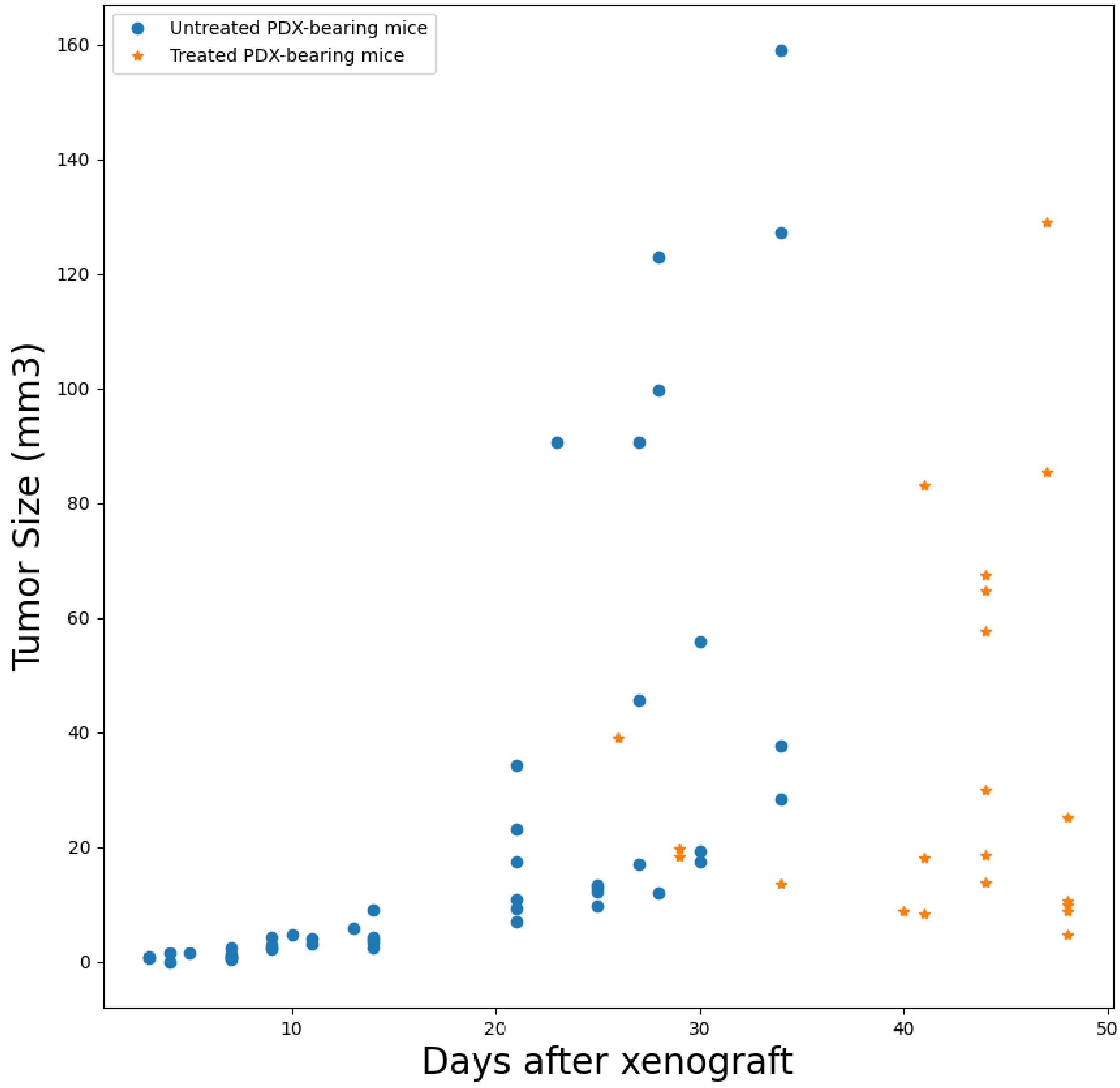

Supplement: Supplementary file 1 [file metabolites-14-00448-s001.zip › metabolites-3012406-supplementary.pdf]
